# The chromosome-level genome of *Cherax quadricarinatus*

**DOI:** 10.1038/s41597-023-02124-z

**Published:** 2023-04-17

**Authors:** Honglin Chen, Rui Zhang, Feng Liu, Changwei Shao, Fangfang Liu, Weidong Li, Jindong Ren, Baolong Niu, Haipeng Liu, Bao Lou

**Affiliations:** 1 State Key Laboratory for Managing Biotic and Chemical Threats to the Quality and Safety of Agro-products, Institute of Hydrobiology, Zhejiang Academy of Agricultural Sciences, Hangzhou, China; 2 BGI-Qingdao, Qingdao, China; 3 Key Lab of Sustainable Development of Marine Fisheries, Ministry of Agriculture and Rural Affairs, Yellow Sea Fisheries Research Institute, Chinese Academy of Fishery Sciences, 266072 Qingdao, Shandong China; 4 State Key Laboratory of Marine Environmental Science; State-Province Joint Engineering Laboratory of Marine Bioproducts and Technology; College of Ocean and Earth Sciences, Xiamen University; Xiamen, 361102 Fujian, China

**Keywords:** Genome evolution, DNA sequencing

## Abstract

Red claw crayfish (*Cherax quadricarinatus*) is an aquatic crustacean with considerable potential for the commercial culture and an ideal model for studying the mechanism of sex determination. To provide better genomic resources, we assembled a chromosome-level genome with a size of 5.26 Gb and contig N50 of 144.33 kb. Nearly 90% of sequences were anchored to 100 chromosomes, which represents the high-quality crustacean genome with the largest number of chromosomes ever reported. The genome contained 78.69% repeat sequences and 20,460 protein-coding genes, of which 82.40% were functionally annotated. This chromosome-scale genome would be a valuable reference for assemblies of other complex genomes and studies of evolution in crustaceans.

## Background & Summary

Crustaceans are a diverse and ancient group of arthropods^1^, and are not only essential components of the marine and freshwater environments, but also an interesting model for the study of evolutionary biology and developmental biology. However, due to the high complexity, assembly of complete and exact crustacean genomes is difficult, let alone genomes at the chromosome level^2^.

*Cherax quadricarinatus*, also known as the red claw crayfish, is a large tropical freshwater crustacean with significant commercial interest for global aquaculture^3^. Intersexuality appears relatively widespread throughout gonochoristic crustaceans and has been reported in several crayfish species^4^. In red claw crayfish, the intersex individuals undergo a dramatic morphological and physiological sex shift, which makes it a fascinate model to study the mechanisms underlying sex determination and differentiation of crustacean. Although a genome of this species has been reported previously, with uncomplete and fragmental genome assembly (assembled genome size, 3.24 Gb and Contig N50, 33 kb), it still prevents many studies from going deep^5^. Here, we *de novo* assembled a chromosome-level genome of red claw crayfish with the assembled genome size of 5.26 Gb and contig N50 of 144,316 bp. This high-quality genome would enrich the genomic resources of crustaceans and provides basic data for further genome-wide selective breeding.

## Methods

### Sample collection and genomic sequencing

All samples used in this study were from a healthy male adult red claw crayfish farmed in Honghai Co., LTD., Zhejiang, China. Fresh muscle and haemolymph were used for whole genomic sequencing and Hi-C sequencing, respectively. Seven tissues including muscle, intestine, eyestalk, hepatopancreas, gills, stomach, and antennal gland were used for transcriptomic sequencing. Isolation of DNA/RNA, construction of libraries and genomic sequencing were carried out according to protocols from https://www.protocols.io/widgets/doi?uri=dx.doi.org/10.17504/protocols.io.bs8inhue.

For whole genomic sequencing (WGS), the genomic DNA was sonicated into ~250 bp fragments that used to build the 100 bp paired-end (PE100) sequencing library. The library was then sequenced on the BGISEQ-500 platform and generated 280.51 Gb raw data, which covered ~58X of the estimated genome (Table 1).

**Table 1:** Statistics of sequencing data.

Types	Sample	Raw reads (Gb)	Clean data (Gb)
PE100 DNA	Muscle	280.51	238.09
20 kb PacBio CLR	Muscle	568.55	
PE100 Hi-C	Hemolymph	542.71	
PE100 RNA	Intestines	15.79	6.91
	Antennal Gland	25.41	11.06
	Hepatopancreas	18.50	10.51
	Muscle	20.17	8.33
	Gill	16.30	7.05
	Stomach	14.81	7.12
	Eyestalk	25.98	13.69

For PacBio Continuous Long Reads (CLR) sequencing, seven sequencing libraries were constructed using ~20Kb high-quality molecular DNA fragments. All libraries were sequenced on the PacBio Sequel II platform, which generated 568.55 Gb raw data with an N50 of 17,393 bp (Table 1).

For the construction of Hi-C library, DNA was fixed with formaldehyde solution and isolated from nuclei, and digested with MboI, the digested fragments were labeled with biotinylated nucleotides. Eight libraries were sequenced on the BGISEQ-500 platform and produced a total of 542.71 Gb raw data, which covered ~105X of the estimated genome (Table 1).

Seven RNA libraries were constructed according to the protocols and sequenced on the BGISEQ-500 platform, generating a total of 136.96 Gb raw data (Table 1).

### Genome survey

**Fig. 1:**
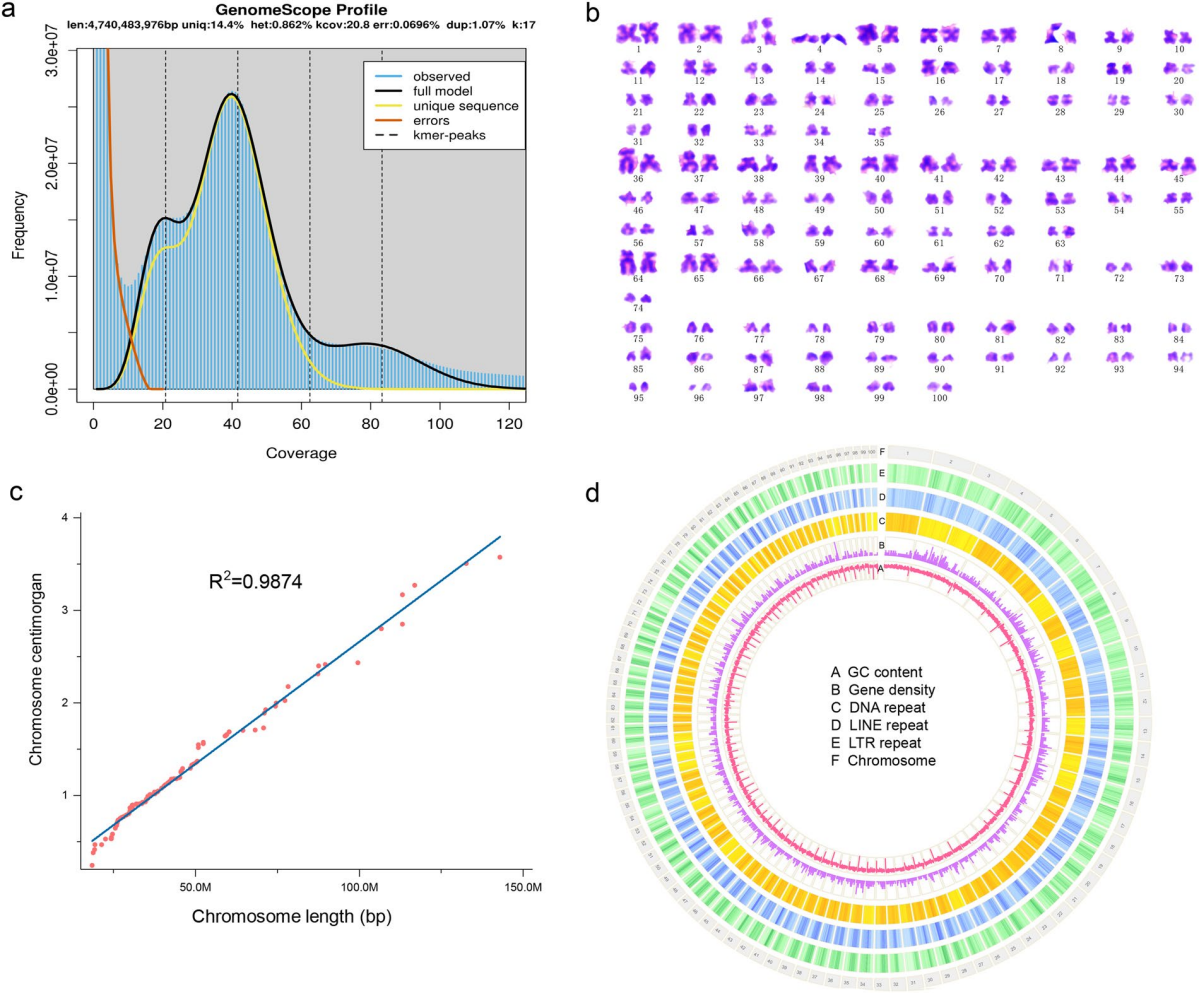
Genome assembly of the red claw crayfish. (**a**) The 17-mer analysis of the genome. (**b**) The karyotypic analysis. The karyotype formula of the male is n = 100 = 36 m + 33 sm + 14 st + 17 t. (**c**) The linear regression analysis between sequence length and physical length of chromosomes. (**d**) Genomic features.

Raw PE100 reads were firstly filtered by SOAPnuke (v1.6.5)^6^ with parameters of “–M 1 –d –A 0.4 –n 0.05 –l 10 –q 0.4 –Q 2 –G –5 0”, and 240 Gb clean data were retained (Table 1). Then Jellyfish (v2.2.6)^7^ was used to count k-17mers and GenomeScope^8^ was used to estimate the size, heterozygosity, and repetitive sequences of the genome at 4.74 Gb, 0.86% and 85.6%, respectively (Fig. 1a).

### Chromosome karyotyping

The number and length of chromosomes in red claw crayfish were obtained by karyotyping experiment using 15 male adults, according to the published pipeline^9^. Chromosomes were measured using Adobe Photoshop CS6 measurement tools under a magnification of 600 × . The chromosome pairs were classified following the nomenclature of Levan (1964)^10^ into m = metacentric (long arm/short arm (r) = 1–1.7), sm = submetacentric (r = 1.7–3), st = subtelocentric (r = 3–7), and a = acrocentric (r > 7). The karyotype formula of the male red claw crayfish is n = 100 = 36 m + 33 sm + 14 st + 17 t (Fig. 1b), and the arm lengths data were listed in Supplementary Table 1.

### Genome assembly

Reads longer than 5 kb were kept from raw Pacbio CLR reads and corrected by Canu (v1.5)^11^, based on which the draft genome was assembled by Wtdbg2^12^ with parameters of “-p 21 -E 2 -S 4 -s 0.05 -L 5000 -X 40”. The draft genome was further polished by Pilon^13^ using clean PE100 reads with default parameters, giving an assembly with the size of 5.26 Gb and the contig N50 of 144.33 kb (Table 2).

**Table 2:** Summary of the genome assembly of red claw crayfish.

	PacBio		Hi-C	
	Scaffold	Contig	Scaffold	Contig
Total number	99,922	100,361	46,864	100,373
Total length of (bp)	5,229,209,719	5,229,209,280	5,255,744,719	5,229,209,280
Gap number (bp)	439	0	26,535,439	0
Average length (bp)	52,333	52,104,00	112,149	52,097,77
N50 Length (bp)	145,977	144,333	45,061,517	144,316
N90 Length (bp)	19,668	19,628	56,367	19,628
Maximum length (bp)	2,570,330	2,570,330	142,949,047	2,570,330
Minimum length (bp)	638	76	638	76
GC content (%)	42.21%	42.21%	42.21%	42.21%

**Fig. 2:**
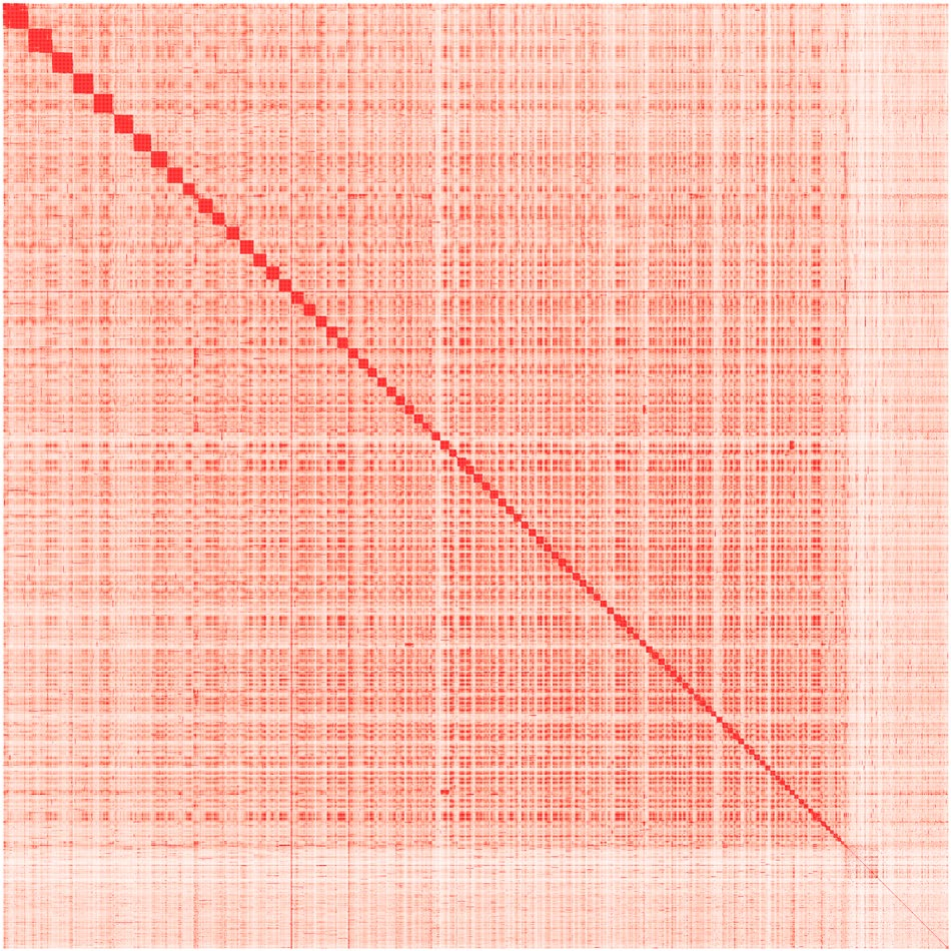
The chromosome matrix heatmap.

Based on the polished genome, 84.34 Gb Hi-C data were validated through quality control by Hi-C-Pro (v. 2.8.0)^14^, which were then applied for chromosomal reconstruction by Juicer (v1.5)^15^ and 3D-DNA (3D-de novo assembly)^16^. To get more precise chromosomes, we manually made some adjustments according to the chromosomal interaction heatmap by Juicebox^17^ (Fig. 2). Finally, a total of 4.70 Gb sequences were anchored to 100 chromosomes, of which the longest is 142.95 Mb and the shortest is 18.54 Mb (Supplementary Table 2). The linear regression analysis of karyotyping and assembly showed a high correlation (*R*^2^ = 0.9874) between the physical length and sequence length of 100 chromosomes (Fig. 1c), indicating the high-quality crustacean genome with the largest number of chromosomes ever reported.

#### Repeat annotation

**Fig. 3:**
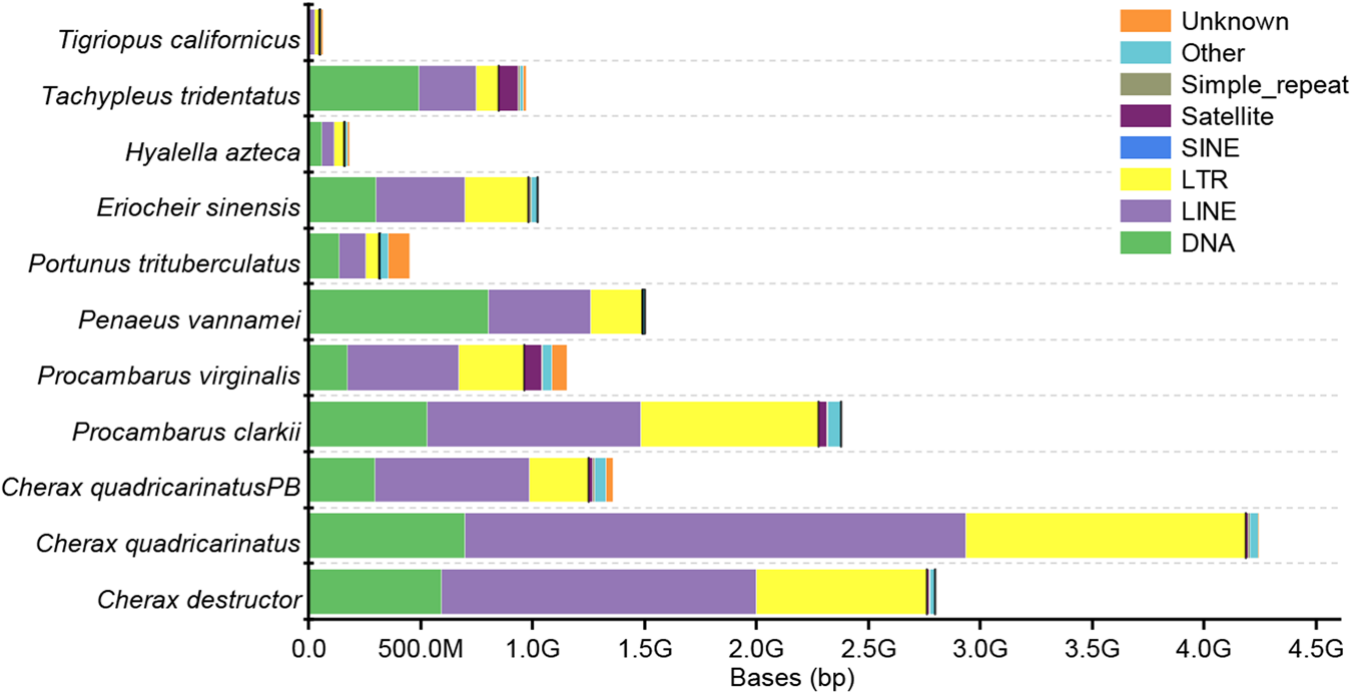
Composition of the major TEs among 11 crustacean species.

Based on aligning the genome to the Repbase library by TRF (v.4.09)^18^, repetitive sequences were predicted by RepeatMasker (v. 3.3.0) and RepeatProteinMask (v. 3.3.0)^19^. In addition, transposable elements (TEs) were constructed and RepeatModeler (v1.0.8)^20^ (Table 3). All the above results together showed that red claw crayfish contains 78.69% repetitive sequences, among which TEs were most abundant (3,482 Mb) (Fig. 3, Table 4). Compared with other decapod crustaceans, the proportion of TES in crayfish was generally much higher.

**Table 3:** Summary of repetitive sequences.

Type	Repeat Size(bp)	% of genome
Tandem Repeat Finder	1,188,877,157	22.621
RepeatMasker	445,491,828	8.476
RepeatProteinMask	626,288,466	11.916
*De novo*	3,479,664,132	66.207
Total	4,135,818,061	78.691

**Table 4:** Summary of different TE repeat sequences.

Type	Repbase TEs		Protein TEs		Denovo TEs		Combined TEs	
	Length(bp)	% of genome	Length(bp)	% of genome	Length(bp)	% of genome	Length(bp)	% of genome
DNA	246,065,669	4.68	21,028,400	0.40	519,239,284	9.88	697,212,505	13.27
LINE	197,906,764	3.77	552,300,634	10.51	2,046,408,761	38.94	2,238,906,979	42.60
SINE	6,813,035	0.13	0	0	2,033,568	0.04	8,812,197	0.17
LTR	54,064,213	1.03	53,065,444	1.01	1,213,361,102	23.09	1,251,832,766	23.82
Other	253,460	0.01	0	0	0	0	253,460	0.01
Unknown	0	0	0	0	8,011,372	0.15	8,011,372	0.15
Total	445,491,828	8.48	626,288,466	11.92	3,436,709,566	65.39	3,481,829,970	66.25

Note: TEs, transposable elements; LINE, long interspersed nuclear elements; SINE, short interspersed nuclear elements; LTR, long terminal repeats.

### Gene prediction

For homology-based gene prediction, the encoded protein sequences of six crustacean species include *Cherax quadricarinatus* (previous version), *Eriocheir sinensis*, *Hyalella azteca*, *Macrobrachium nipponense*, *Penaeus vannamei*, and *Procambarus virginalis* were aligned with the genomic sequence of red claw crayfish using BLAST^20^ and Genewise^21^ with default parameters. Augustus (v3.2.3)^22^ and Genscan^23^ were used for *de novo* gene prediction^24^. RNA reads were mapped to the genome by HISAT2 (v2.1.0)^25^ and gene structure were predicted by Stringtie (v1.2.2)^26^. Meanwhile, transcriptome was *de novo* assembled by Trinity (v2.1.1)^27^ and splicing variations were identified by PASApipeline (v2.4.1)^28^. EVidenceModeler (v1.1)^29^ was applied to integrate the above evidence and a total of 20,460 protein-coding genes were predicted, with average gene length and exon number per gene of 40,182.55 bp and 6.5, respectively (Tables 5, 6).

**Table 5:** Statistical results of gene structure prediction.

	Gene set	Gene number	Gene length(bp)	CDS number	Intron length(bp)	Exon length(bp)	Exon per gene	BUSCO
Homolog	*C. quadricarinatus*	37,558	25,503.02	665.20	11,214.81	206.92	3.21	C:76.50%
	*E. sinensis*	10,669	29,723.88	913.70	8,792.97	213.66	4.28	C:51.60%
	*H. azteca*	6,232	47,229.48	1,022.85	11,887.37	209.30	4.89	C:45.50%
	*M. nipponense*	15,693	56,965.30	913.50	20,215.13	242.13	3.77	C:37.30%
	*P. vannamei*	14,642	128,560.17	1,217.39	31,515.52	241.52	5.04	C:67.30%
	*P. virginalis*	22,924	42,488.16	667.11	22,422.71	232.84	2.87	C:61.70%
Denovo	Augustus	197,737	13,764.73	1,220.68	7,260.74	447.52	2.73	C:65.30%
	Genscan	296,975	9,888.34	1,255.71	2,914.62	316.95	3.96	C:66.30%
Transcriptome	Hisat + Stringtie	78,739	36,776.39	841.27	10,097.69	269.31	3.12	C:82.60%
EVM		234,118	12,363.55	1,186.88	4,680.45	350.32	3.39	C:80.40%
Final		20,460	40,182.55	1,753.24	6,992.06	269.89	6.50	C:88.30%

**Table 6:** BUSCO evaluation of gene annotation in red claw crayfish.

Type	Percentage
Complete BUSCOs	88.3% (941)
Complete Single-Copy BUSCOs	86.2% (919)
Complete Duplicated BUSCOs	2.1% (22)
Fragmented BUSCOs	5.7% (61)
Missing BUSCOs	6.0% (64)
Total	100% (1066)

These genes were then functionally annotated through BLAST against NCBI non-redundant proteins (NR), TrEMBL, Gene Ontology (GO), SwissProt, and Kyoto Encyclopedia of Genes and Genomes (KEGG) protein databases. Finally, 16,859 genes accounting for 82.40% of the total were successfully annotated with at least one public functional database (Table 7).

**Table 7:** Summary of gene annotation in red claw crayfish.

Database	Number	Percentage
Total	20,460	100%
NR	15,959	78.00%
Swissprot-Annotated	12,318	60.21%
KEGG-Annotated	13,570	66.32%
TrEMBL-Annotated	16,303	79.68%
Interpro-Annotated	12,536	61.27%
GO-Annotated	8,861	43.31%
Overall	16,859	82.40%

The tRNAscan-SE^30^ was used to annotate the tRNAs based on annotated features such as isotype, anticodon, and tRNAscan-SE bit score. The rRNA sequences were annotated from homologous references in close species. MiRNAs and snRNAs were predicted by the INFERNAL^31^ based on the covariance model of the Rfam database. Totally 6,954 non-coding RNAs were predicted, including 25 miRNA, 1,448 rRNA, 5,023 tRNA and 458 snRNA genes (Table 8).

**Table 8:** Statistics of annotated non-coding RNAs.

Type		Copy	Average length(bp)	Total length(bp)	% of genome
miRNA		25	77.64	1,941	0.0000
tRNA		5023	71.63	359,796	0.0068
rRNA	rRNA	724	138.72	100,431	0.0019
	18 S	225	162.96	36,665	0.0007
	28 S	374	158.15	59,147	0.0011
	5.8 S	17	52.06	885	0.0000
	5 S	108	34.57	3,734	0.0001
	8 S	0	0	0	0
snRNA	snRNA	229	137.39	31,462	0.0006
	CD-box	1	199.00	199	0.0000
	HACA-box	0	0	0	0
	splicing	228	137.12	31,263	0.0006
	scaRNA	0	0	0	0

## Data Records

The genomic WGS sequencing data were deposited in the SRA at NCBI SRR22412649^32^, SRR22412641^33^.

The genomic PacBio sequencing data were deposited in the SRA at NCBI SRR22412654^34^.

The transcriptomic sequencing data were deposited in the SRA at NCBI SRR22412651^35^, SRR22412652^36^, SRR22412653^37^, SRR22412637^38^, SRR22412638^39^, SRR22412639^40^, SRR22412640^41^.

The Hi-C sequencing data were deposited in the SRA at NCBI SRR22412642^42^, SRR22412643^43^, SRR22412644^44^, SRR22412645^45^, SRR22412646^46^, SRR22412647^47^, SRR22412648^48^, SRR22412650^49^.

The final chromosome assembly was deposited in GenBank at NCBI JAPQEV000000000^50^.

The genome annotation file is available in figshare^51^.

## Technical Validation

The quality and quantity of total DNA was checked using agarose gel electrophoresis, and the concentration was determined using a NanoDrop 2000 spectrophotometer. RNA integrity was evaluated using an Agilent 2100 Bioanalyzer (Agilent Technologies, CA, USA). The sample used in our study had an RNA integrity number (RIN) larger than 8. To further assess the quality of the genome, clean PE100 reads were aligned back to the genome by BWA^52^, showing the mapping rate as high as 99.03%. The depth and GC content were also statistically analyzed within a 10Kb sliding window. Moreover, 85.7% completed and 6.2% fragmented BUSCOs^53^ (Benchmarking Universal Single-Copy Orthologs, v4.0) in arthropoda_odb9 database were identified, which showed a noticeable improvement than the previous version (81.3%).

## Supplementary information

supplementary table

## Data Availability

No specific code was developed in this work. The parameters of all commands and pipelines used for data processing are described in the Methods section. If no detailed parameters are mentioned for a software, the default parameters were used, as suggested by the developer.
