# Publisher Correction: The chromosome-level genome of *Cherax quadricarinatus*

**DOI:** 10.1038/s41597-023-02186-z

**Published:** 2023-05-23

**Authors:** Honglin Chen, Rui Zhang, Feng Liu, Changwei Shao, Fangfang Liu, Weidong Li, Jindong Ren, Baolong Niu, Haipeng Liu, Bao Lou

**Affiliations:** 1 State Key Laboratory for Managing Biotic and Chemical Threats to the Quality and Safety of Agro-products, Institute of Hydrobiology, Zhejiang Academy of Agricultural Sciences, Hangzhou, China; 2 BGI-Qingdao, Qingdao, China; 3 Key Lab of Sustainable Development of Marine Fisheries, Ministry of Agriculture and Rural Affairs, Yellow Sea Fisheries Research Institute, Chinese Academy of Fishery Sciences, 266072 Qingdao, Shandong China; 4 State Key Laboratory of Marine Environmental Science; State-Province Joint Engineering Laboratory of Marine Bioproducts and Technology; College of Ocean and Earth Sciences, Xiamen University; Xiamen, 361102 Fujian, China

**Keywords:** Genome evolution, DNA sequencing

Correction to: *Scientific Data* 10.1038/s41597-023-02124-z, published online 17 April 2023

In this article the affiliation details for Rui Zhang were incorrectly given as Xiamen University but should have been BGI-Qingdao; the affiliation details for Weidong Li were incorrectly given as BGI-Qingdao but should have been Xiamen University; and the affiliation details for Haipeng Liu were incorrectly given as BGI-Qingdao but should have been Xiamen University. The original article has been corrected.

