# Supplementary material for: The chromosome-level genome of Cherax quadricarinatus
Source: Sci Data. 2023 Apr 17;10:215. doi: 10.1038/s41597-023-02124-z (PMC10106460; doi:10.1038/s41597-023-02124-z)
Supplement: Supplementary file 1 — supplementary table [file 41597_2023_2124_MOESM1_ESM.docx]

Supplementary Table 1. Characteristics of the karyotype of red claw crayfish.

| Chromosome pair no. | Long arm  (μm) | Short arm  (μm) | Total length  (μm) | Centromeric index | Relative length（%） | Classification |
| --- | --- | --- | --- | --- | --- | --- |
| 1 | 2.036 | 1.468 | 3.504 | 0.419 | 2.793% | Metacentric |
| 2 | 1.773 | 1.076 | 2.849 | 0.378 | 2.270% | Metacentric |
| 3 | 1.597 | 1.202 | 2.799 | 0.429 | 2.231% | Metacentric |
| 4 | 1.377 | 1.034 | 2.411 | 0.429 | 1.922% | Metacentric |
| 5 | 1.263 | 1.136 | 2.399 | 0.474 | 1.912% | Metacentric |
| 6 | 1.185 | 0.839 | 2.024 | 0.414 | 1.613% | Metacentric |
| 7 | 1.166 | 0.720 | 1.885 | 0.382 | 1.502% | Metacentric |
| 8 | 0.927 | 0.759 | 1.685 | 0.450 | 1.343% | Metacentric |
| 9 | 0.998 | 0.657 | 1.655 | 0.397 | 1.319% | Metacentric |
| 10 | 0.861 | 0.782 | 1.642 | 0.476 | 1.309% | Metacentric |
| 11 | 0.912 | 0.602 | 1.514 | 0.397 | 1.206% | Metacentric |
| 12 | 0.780 | 0.588 | 1.367 | 0.430 | 1.090% | Metacentric |
| 13 | 0.761 | 0.593 | 1.354 | 0.438 | 1.079% | Metacentric |
| 14 | 0.799 | 0.528 | 1.327 | 0.398 | 1.057% | Metacentric |
| 15 | 0.703 | 0.491 | 1.193 | 0.411 | 0.951% | Metacentric |
| 16 | 0.710 | 0.468 | 1.178 | 0.397 | 0.939% | Metacentric |
| 17 | 0.672 | 0.467 | 1.139 | 0.410 | 0.908% | Metacentric |
| 18 | 0.628 | 0.506 | 1.134 | 0.446 | 0.903% | Metacentric |
| 19 | 0.588 | 0.446 | 1.034 | 0.431 | 0.824% | Metacentric |
| 20 | 0.573 | 0.436 | 1.009 | 0.432 | 0.804% | Metacentric |
| 21 | 0.544 | 0.462 | 1.006 | 0.459 | 0.802% | Metacentric |
| 22 | 0.552 | 0.437 | 0.989 | 0.442 | 0.788% | Metacentric |
| 23 | 0.557 | 0.411 | 0.967 | 0.425 | 0.771% | Metacentric |
| 24 | 0.538 | 0.366 | 0.904 | 0.405 | 0.721% | Metacentric |
| 25 | 0.552 | 0.352 | 0.904 | 0.390 | 0.720% | Metacentric |
| 26 | 0.537 | 0.339 | 0.876 | 0.387 | 0.698% | Metacentric |
| 27 | 0.517 | 0.348 | 0.865 | 0.402 | 0.689% | Metacentric |
| 28 | 0.500 | 0.363 | 0.863 | 0.420 | 0.687% | Metacentric |
| 29 | 0.489 | 0.353 | 0.842 | 0.419 | 0.671% | Metacentric |
| 30 | 0.442 | 0.356 | 0.797 | 0.446 | 0.635% | Metacentric |
| 31 | 0.418 | 0.333 | 0.751 | 0.444 | 0.598% | Metacentric |
| 32 | 0.369 | 0.326 | 0.695 | 0.469 | 0.554% | Metacentric |
| 33 | 0.391 | 0.283 | 0.674 | 0.419 | 0.537% | Metacentric |
| 34 | 0.303 | 0.276 | 0.579 | 0.476 | 0.461% | Metacentric |
| 35 | 0.290 | 0.236 | 0.526 | 0.448 | 0.419% | Metacentric |
| 36 | 0.240 | 0.174 | 0.414 | 0.420 | 0.330% | Metacentric |
| 37 | 2.611 | 0.961 | 3.572 | 0.269 | 2.847% | Submetacentric |
| 38 | 2.361 | 0.909 | 3.270 | 0.278 | 2.606% | Submetacentric |
| 39 | 2.220 | 0.947 | 3.167 | 0.299 | 2.524% | Submetacentric |
| 40 | 1.648 | 0.661 | 2.309 | 0.286 | 1.840% | Submetacentric |
| 41 | 1.301 | 0.696 | 1.997 | 0.349 | 1.592% | Submetacentric |
| 42 | 1.209 | 0.517 | 1.725 | 0.299 | 1.375% | Submetacentric |
| 43 | 1.135 | 0.564 | 1.699 | 0.332 | 1.354% | Submetacentric |
| 44 | 1.097 | 0.460 | 1.557 | 0.295 | 1.241% | Submetacentric |
| 45 | 0.894 | 0.355 | 1.249 | 0.284 | 0.995% | Submetacentric |
| 46 | 0.840 | 0.423 | 1.263 | 0.335 | 1.006% | Submetacentric |
| 47 | 0.820 | 0.467 | 1.287 | 0.363 | 1.026% | Submetacentric |
| 48 | 0.807 | 0.326 | 1.133 | 0.288 | 0.903% | Submetacentric |
| 49 | 0.774 | 0.373 | 1.147 | 0.325 | 0.914% | Submetacentric |
| 50 | 0.774 | 0.302 | 1.076 | 0.280 | 0.857% | Submetacentric |
| 51 | 0.773 | 0.417 | 1.190 | 0.350 | 0.948% | Submetacentric |
| 52 | 0.755 | 0.422 | 1.177 | 0.358 | 0.938% | Submetacentric |
| 53 | 0.755 | 0.420 | 1.175 | 0.357 | 0.936% | Submetacentric |
| 54 | 0.753 | 0.344 | 1.097 | 0.314 | 0.874% | Submetacentric |
| 55 | 0.755 | 0.278 | 1.033 | 0.269 | 0.823% | Submetacentric |
| 56 | 0.732 | 0.291 | 1.023 | 0.284 | 0.815% | Submetacentric |
| 57 | 0.705 | 0.332 | 1.037 | 0.320 | 0.827% | Submetacentric |
| 58 | 0.638 | 0.263 | 0.901 | 0.292 | 0.718% | Submetacentric |
| 59 | 0.631 | 0.278 | 0.908 | 0.306 | 0.724% | Submetacentric |
| 60 | 0.626 | 0.301 | 0.926 | 0.325 | 0.738% | Submetacentric |
| 61 | 0.615 | 0.250 | 0.864 | 0.289 | 0.689% | Submetacentric |
| 62 | 0.613 | 0.328 | 0.941 | 0.348 | 0.750% | Submetacentric |
| 63 | 0.606 | 0.289 | 0.895 | 0.323 | 0.713% | Submetacentric |
| 64 | 0.575 | 0.227 | 0.802 | 0.283 | 0.639% | Submetacentric |
| 65 | 0.523 | 0.261 | 0.784 | 0.332 | 0.625% | Submetacentric |
| 66 | 0.509 | 0.250 | 0.759 | 0.329 | 0.605% | Submetacentric |
| 67 | 0.488 | 0.281 | 0.769 | 0.365 | 0.613% | Submetacentric |
| 68 | 0.474 | 0.239 | 0.713 | 0.335 | 0.568% | Submetacentric |
| 69 | 0.354 | 0.192 | 0.546 | 0.352 | 0.435% | Submetacentric |
| 70 | 1.912 | 0.521 | 2.433 | 0.214 | 1.939% | Subtelocentric |
| 71 | 1.847 | 0.325 | 2.172 | 0.150 | 1.731% | Subtelocentric |
| 72 | 1.590 | 0.372 | 1.962 | 0.189 | 1.563% | Subtelocentric |
| 73 | 1.450 | 0.254 | 1.704 | 0.149 | 1.358% | Subtelocentric |
| 74 | 1.337 | 0.304 | 1.641 | 0.185 | 1.308% | Subtelocentric |
| 75 | 1.205 | 0.339 | 1.544 | 0.220 | 1.231% | Subtelocentric |
| 76 | 1.043 | 0.293 | 1.336 | 0.219 | 1.065% | Subtelocentric |
| 77 | 0.849 | 0.226 | 1.075 | 0.210 | 0.857% | Subtelocentric |
| 78 | 0.787 | 0.226 | 1.013 | 0.223 | 0.807% | Subtelocentric |
| 79 | 0.721 | 0.237 | 0.958 | 0.247 | 0.763% | Subtelocentric |
| 80 | 0.740 | 0.185 | 0.925 | 0.200 | 0.737% | Subtelocentric |
| 81 | 0.709 | 0.195 | 0.904 | 0.216 | 0.721% | Subtelocentric |
| 82 | 0.711 | 0.192 | 0.903 | 0.212 | 0.719% | Subtelocentric |
| 83 | 0.682 | 0.207 | 0.889 | 0.233 | 0.708% | Subtelocentric |
| 84 | 1.923 | 0 | 1.923 | 0 | 1.532% | Telocentric |
| 85 | 1.571 | 0 | 1.571 | 0 | 1.252% | Telocentric |
| 86 | 1.290 | 0 | 1.290 | 0 | 1.028% | Telocentric |
| 87 | 1.113 | 0 | 1.113 | 0 | 0.887% | Telocentric |
| 88 | 1.046 | 0 | 1.046 | 0 | 0.834% | Telocentric |
| 89 | 0.910 | 0 | 0.910 | 0 | 0.725% | Telocentric |
| 90 | 0.827 | 0 | 0.827 | 0 | 0.659% | Telocentric |
| 91 | 0.868 | 0 | 0.868 | 0 | 0.691% | Telocentric |
| 92 | 0.754 | 0 | 0.754 | 0 | 0.601% | Telocentric |
| 93 | 0.731 | 0 | 0.731 | 0 | 0.583% | Telocentric |
| 94 | 0.643 | 0 | 0.643 | 0 | 0.513% | Telocentric |
| 95 | 0.529 | 0 | 0.529 | 0 | 0.421% | Telocentric |
| 96 | 0.529 | 0 | 0.529 | 0 | 0.421% | Telocentric |
| 98 | 0.467 | 0 | 0.467 | 0 | 0.372% | Telocentric |
| 97 | 0.464 | 0 | 0.464 | 0 | 0.370% | Telocentric |
| 99 | 0.380 | 0 | 0.380 | 0 | 0.302% | Telocentric |
| 100 | 0.244 | 0 | 0.244 | 0 | 0.194% | Telocentric |

Supplementary Table 2. Summary of chromosome lengths based on Hi-C-assisted assembly.

| Chromosome | Length (bp) | Ratio | Chromosome | Length (bp) | Ratio |
| --- | --- | --- | --- | --- | --- |
| chr1 | 142,949,047 | 2.72% | chr51 | 38,569,588 | 0.73% |
| chr2 | 132,683,543 | 2.52% | chr52 | 38,207,155 | 0.72% |
| chr3 | 116,934,865 | 2.22% | chr53 | 37,615,761 | 0.72% |
| chr4 | 113,186,082 | 2.15% | chr54 | 37,630,315 | 0.72% |
| chr5 | 113,256,706 | 2.15% | chr55 | 37,648,981 | 0.69% |
| chr6 | 06,814,695 | 2.03% | chr56 | 36,370,656 | 0.69% |
| chr7 | 99,615,393 | 1.90% | chr57 | 36,160,939 | 0.68% |
| chr8 | 89,689,027 | 1.71% | chr58 | 35,546,502 | 0.68% |
| chr9 | 87,686,776 | 1.67% | chr59 | 35,549,958 | 0.67% |
| chr10 | 87,392,546 | 1.66% | chr60 | 35,067,005 | 0.66% |
| chr11 | 78,361,451 | 1.49% | chr61 | 34,947,312 | 0.66% |
| chr12 | 77,386,932 | 1.47% | chr62 | 34,827,483 | 0.65% |
| chr13 | 74,651,552 | 1.42% | chr63 | 33,899,955 | 0.64% |
| chr14 | 74,449,604 | 1.42% | chr64 | 33,673,000 | 0.63% |
| chr15 | 71,509,238 | 1.36% | chr65 | 33,252,750 | 0.63% |
| chr16 | 71,045,516 | 1.35% | chr66 | 32,863,844 | 0.62% |
| chr17 | 70,847,130 | 1.35% | chr67 | 32,333,128 | 0.61% |
| chr18 | 68,244,828 | 1.30% | chr68 | 31,990,470 | 0.61% |
| chr19 | 64,574,666 | 1.23% | chr69 | 31,903,288 | 0.61% |
| chr20 | 60,327,206 | 1.15% | chr70 | 31,858,498 | 0.59% |
| chr21 | 59,657,907 | 1.14% | chr71 | 30,722,648 | 0.58% |
| chr22 | 59,299,090 | 1.13% | chr72 | 30,874,960 | 0.59% |
| chr23 | 59,087,076 | 1.12% | chr73 | 30,729,222 | 0.58% |
| chr24 | 52,476,258 | 1.00% | chr74 | 30,614,505 | 0.58% |
| chr25 | 52,418,708 | 1.00% | chr75 | 30,141,103 | 0.57% |
| chr26 | 50,987,147 | 0.97% | chr76 | 30,081,197 | 0.57% |
| chr27 | 50,911,439 | 0.97% | chr77 | 30,170,993 | 0.57% |
| chr28 | 50,666,775 | 0.96% | chr78 | 30,052,949 | 0.57% |
| chr29 | 50,230,587 | 0.96% | chr79 | 29,816,050 | 0.57% |
| chr30 | 49,462,227 | 0.94% | chr80 | 29,641,722 | 0.56% |
| chr31 | 49,423,845 | 0.93% | chr81 | 28,872,412 | 0.55% |
| chr32 | 48,884,978 | 0.92% | chr82 | 28,170,810 | 0.54% |
| chr33 | 48,508,739 | 0.88% | chr83 | 27,619,271 | 0.53% |
| chr34 | 46,125,467 | 0.87% | chr84 | 27,432,344 | 0.52% |
| chr35 | 45,740,544 | 0.87% | chr85 | 27,076,919 | 0.52% |
| chr36 | 45,600,167 | 0.86% | chr86 | 26,402,419 | 0.50% |
| chr37 | 45,256,275 | 0.86% | chr87 | 26,270,035 | 0.50% |
| chr38 | 45,061,517 | 0.84% | chr88 | 26,163,968 | 0.50% |
| chr39 | 44,214,542 | 0.83% | chr89 | 25,984,450 | 0.49% |
| chr40 | 43,435,958 | 0.81% | chr90 | 25,569,382 | 0.49% |
| chr41 | 42,728,638 | 0.80% | chr91 | 24,743,527 | 0.47% |
| chr42 | 42,196,477 | 0.80% | chr92 | 24,458,011 | 0.47% |
| chr43 | 41,956,719 | 0.80% | chr93 | 24,316,755 | 0.46% |
| chr44 | 41,865,233 | 0.79% | chr94 | 24,381,195 | 0.46% |
| chr45 | 41,663,554 | 0.78% | chr95 | 22,620,991 | 0.43% |
| chr46 | 40,861,055 | 0.77% | chr96 | 21,469,115 | 0.41% |
| chr47 | 40,418,686 | 0.76% | chr97 | 19,375,509 | 0.37% |
| chr48 | 39,745,675 | 0.76% | chr98 | 19,253,805 | 0.37% |
| chr49 | 39,748,171 | 0.74% | chr99 | 18,801,782 | 0.36% |
| chr50 | 38,637,708 | 0.73% | chr100 | 18,541,734 | 0.35% |
